# High immersion/escapism motivation makes gaming disorder risk less dependent of playtime among highly engaged male gamers

**DOI:** 10.3389/fpsyt.2024.1443091

**Published:** 2024-09-25

**Authors:** Patrycja Kiszka, Agnieszka Strojny, Paweł Strojny

**Affiliations:** ^1^ Doctoral School in the Social Sciences, Jagiellonian University, Kraków, Poland; ^2^ Institute of Applied Psychology, Faculty of Management and Social Communication, Jagiellonian University, Kraków, Poland

**Keywords:** gaming disorder, gaming time, escapism, immersion, gaming motivation

## Abstract

In the realm of gaming-related concerns, the relationship between gaming time (GT) and gaming disorder (GD) remains an intriguing and complex subject. Although increased GT is not a reliable predictor of GD risk, the circumstances under which this relationship strengthens or weakens remain relatively unknown. This study explores the roles of immersion/escapism motive (IEM) and GT in the context of GD among highly engaged gamers (N = 294), each dedicating a minimum of 20 hours weekly to gaming. The findings confirm that IEM significantly moderates the relationship between GT and GD in the male sample. Specifically, low and moderate levels of IEM result in a stronger relationship between GT and GD. In the case of women, the effect was not significant. These findings suggest the importance of comprehensive assessments of gaming motivations when addressing gaming-related issues, particularly in GD research. Moreover, they emphasize the value of adopting a complex approach to comprehending the development of problematic gaming behaviors.

## 1 Introduction

The advancements in digital entertainment opened up a new realm of mental health concerns. Among these is gaming disorder (GD), officially recognized in 2018 by the World Health Organization and included in the 11^th^ Revision of the International Classification of Diseases (1). Recognizing GD as a mental health problem has led to increased research aimed at understanding the phenomenon and its associations (e.g., 2–7).

Naturally, gaming time (GT) is considered one of the predictors of GD. However, research indicates that, in contrast to other problematic behaviors of similar nature (gambling, pornography exposure), in the case of GD, the intensity of exposure to a potentially addictive stimulus does not constitute a significant increase in the risk of developing unhealthy behavior patterns as the association between GT and GD has been reported as weak or moderate (8–12). Most gamers do not develop problematic gaming behaviors regardless of GT, and therefore the GT alone is not considered a reliable indicator of GD (12, 13). These findings led to opposition to the recognition of prolonged gaming as the main factor contributing to the development of GD, resulting in the widely cited article by Kiraly et al. (12) with the telling title 'Intense Video Gaming Is Not Essentially Problematic'.

Recent discoveries (14, 15) suggest that one potential reason for GT's poor prediction of GD is the overlooked moderating influence of risk and protective factors on the relationship. It is argued that investigating potential moderators would allow a deeper understanding of GD development by testing the interactions between the variables involved in this mechanism (14, 15). Therefore, it may provide a more nuanced understanding of the conditions under which GT leads to unwanted outcomes and when it does not.

To answer the question of what may constitute a risk factor for GD, one can focus on factors beyond GT that have so far been reported as associated with GD. One of these factors may be gaming motivation. Two literature reviews investigating the relationship between gaming motivations and GD have been published in recent years (16, 17) and both concur that among the various motivations, escapism, defined as playing video games to avoid everyday problems and difficulties (18–20), is most strongly associated with GD. This conclusion is supported by empirical studies (e.g., 21, 22), which have demonstrated moderately strong positive associations between escapism and problematic gaming. Similar findings were reported by Hagström and Kaldo (10), who further introduced the concept of 'negative escapism' in their study. Escapism has also been identified as a mediator of the relationship between psychopathological symptoms/emotion dysregulation and GD (23, 24) suggesting that gamers struggling with depression or anxiety tend to avoid problems and difficulties by escaping into the gaming world, thus favoring their over-involvement, leading to the development of GD.

Moreover, Bäcklund et al. (16) state that, beyond escapism, introjected regulation – defined as a motivation to play in order to feel better or to avoid feeling bad about oneself, and coping – regarding a motivation to play to control negative emotions, were significantly associated with disordered gaming symptoms. Studies subject to the review indicate that introjected regulation (25, 26) and coping (27, 28) are positively associated with disordered gaming, with the strength of these associations ranging from weak to moderate. Research also suggests that heavy gamers exhibit higher introjected regulation scores than light gamers (29). Additionally, fantasy motivation has also been highlighted in the literature as being linked to problematic gaming in both cross-sectional and longitudinal studies (e.g., 30–32). Hence, it can be deduced that the distinct components of immersion/escapism motive (IEM) have been consistently linked to GD. This assertion has further been affirmed by the authors of the Gaming Motivation Inventory (GMI) who suggest that both the IEM and Habit/Boredom motives constitute the highest potential hazard for the onset of GD symptoms among other motives (20).

The relationship between gaming motivations and the risk of GD is a well-researched topic. However, most studies have primarily focused on their direct relationship (e.g., 27, 28, 33–35). In our opinion, framing the issue solely in terms of GD being related to or caused by specific motivations is an oversimplification, as GD cannot develop without the use of games themselves. Therefore, comprehensive models of GD development that consider gaming motivations must also include the act of gaming. We believe that the interaction between GT and IEM is crucial for understanding the development of GD.

The study by Koncz et al. (36), demonstrated that escapism significantly moderates the GT-GD relationship in adolescent males. Our study aims to expand on these findings by including a broader demographic of both adolescent and adult gamers, particularly those highly engaged in gaming. By focusing on committed gamers, our research will help determine whether extensive GT still predicts GD poorly, and if motivations, particularly those perceived as harmful, would make this relationship stronger. Specifically, our hypothesis is that IEM moderates the association between GT and GD, with higher levels of IEM strengthening this relationship. Notably, based on the results obtained by Koncz et al. (36), which indicated differences between males and females in this regard, we decided to separate male and female participants when conducting the analyses.

Examining whether IEM moderates the relationship between GT and GD holds significant promise. While substantial GT alone may not inherently lead to GD, exploring the risk factors is critical in understanding why certain players develop problematic behaviors while others do not. By investigating the conditions under which GT becomes problematic, we can shift the focus from merely GT to addressing the underlying reasons for GD. This shift is crucial, as therapeutic and preventive measures that predominantly emphasize reducing GT might potentially lead to the stigmatization of healthy gaming practices while omitting the key problem.

## 2 Materials and methods

### 2.1 Participants

The survey was conducted in January 2022, via the Internet, using the Qualtrics survey platform (https://qualtrics.com/). The study's URL was shared on gaming-related Polish groups on Facebook as part of a convenience sampling approach. There were two screening questions regarding the minimum age of 13 years and playing video games, and two attention checks in the survey. A total of 1446 Polish-speaking participants took part in the study. Data from 68 were rejected because they did not meet the screening criteria. Additionally, 9 respondents who claimed to spend 24 or more hours per day gaming were not included in the analysis. This resulted in a sample of 1369 participants. The gaming motivation tool used in the study (GMI) was designed based on highly engaged gamers who play at least 20 hours a week (20). Therefore, we decided to apply the same criteria in this study, selecting only highly engaged gamers who play at least 20 hours per week. As a result, the final sample for analysis comprised 294 participants.

### 2.2 Measures

Gaming Disorder Risk was measured with the Gaming Disorder Test (37), Polish adaptation (38). The score can range from 4 to 20 points, with higher scores indicating higher risk of GD. GDT allows researchers to distinguish potentially disordered gamers from non-disordered gamers by checking whether participants meet all four diagnostic criteria included in the individual GDT items, considering responses of '4: Often' or '5: Very often'. However, in this study the distinction was not highlighted in the analyses as, in order to verify the hypothesis, GD was treated as a continuous variable. The GDT shows good internal consistency (Cronbach's alpha = 0.84 to 0.87) and satisfactory construct validity, with good model fit indices from confirmatory factor analysis (37).Cronbach's alpha was 0.77 in the present sample.

Immersion/escapism motive was measured with the Gaming Motivation Inventory (20), Polish translation. The result was calculated as a sum of values of 19 items comprising five factors falling under the immersion/escapism motive: Coping, Escape, Fantasy, Identity, and Introjected Regulation. The scores range from 19 to 133, with higher scores reflecting a greater level of IEM. The GMI is a psychometrically valid tool showing good psychometric properties (20). Cronbach's alphas of the factors within IEM range from 0.75 to 0.89 (20). Overall Cronbach's alpha of IEM was 0.87 in the present sample.

Gaming Time was measured with the Gaming Involvement Scale (15). The average weekly gaming time was calculated by the sum of minutes spent on gaming during average weekdays and average weekend days.

### 2.3 Statistical analysis

Data were analyzed with Imago Pro 9.0 and PROCESS macro (39), version 4.2. Pearson's correlation and moderation analyses were conducted to verify the hypothesis, while linear regression was performed for exploratory purposes.

## 3 Results

We analyzed the data from 294 highly engaged gamers (playing at least 20 hours weekly). The average age of females (N = 92) was 20.8 (*SD* = 5.4) with a range between 13 and 46 years. The average age of males (N = 202) was 22.3 (*SD* = 5.6) with a range between 13 and 44 years. The details and rationale of the selection can be found in the **Supplementary Materials**. Two women and three men from the present study's sample met the criteria of the GDT, qualifying as potentially having GD (37).

In the present study, the effect size was calculated *post hoc* for the regression analysis using the obtained *R^2^* value of 0.15, which resulted in an *f^2^* of 0.176. According to Cohen's criteria, this effect size is considered small to medium. A *post hoc* power analysis revealed that a sample size of 76 participants would be required to achieve a power of 0.95 with this effect size at an alpha level of 0.05. The actual sample size of 294 participants (92 females and 202 males) suggests that the study was adequately powered.

Pearson's correlation analyses showed that in the female sample, there was a moderate positive correlation between IEM and GD (*r* = 0.401, *p* < 0.001), yet the correlation between GT and GD was not statistically significant (*r* = -0.37, *p* = 0.725). Among males, there was a weak positive correlation between IEM and GD (*r* = 0.299, *p* < 0.001) and a weak positive correlation between GT and GD (*r* = 0.229, *p* < 0.01) (**Table 1**).

**Table 1:** Descriptive statistics and Pearson's correlations among independent and dependent variables in the analysis.

Gender	Variable	*M*	*SD*	Min	Max	Gaming Disorder	Gaming Time	Immersion/escapism
Females (*N* = 92)	Gaming Disorder	8.89	3.44	4	18	–	– 0.037	0.401**
	Gaming Time	1731.85	531.70	1200	3500	– 0.037	–	0.183
	Immersion/escapism	76.64	21.78	32	121	0.401**	0.183	–
Males (*N* = 202)	Gaming Disorder	8.62	3.19	4	20	–	0.229*	0.299**
	Gaming Time	1814.90	730.35	1200	7000	0.229*	–	0.062
	Immersion/escapism	74.62	19.43	30	122	0.299**	0.062	–

Gaming time is presented in minutes played per week.

**p < 0.001.

*p < 0.01.

According to the hypothesis regarding males, the interactional influence between GT and IEM on GD was analyzed, as shown in **Table 2**. GT was a significant predictor of GD, *b* = 0.003, *t*(198) = 2.88, *p* < 0.05, whereas IEM alone was not statistically significant as a predictor, *b* = 0.10, *t*(198) = 3.54, *p* = 0.15. However, the overall model including GT and IEM explained 15% of the variance of GD (*F*(3, 198) = 11.76, *p* < 0.001, *R^2^* = 0.15), this is a significant improvement over a simple regression model without IEM as moderator, which explained only 5% of the variance (*F*(1, 200) = 11.11, *p* < 0.01, *R^2^* = 0.05). The interaction term, GT × IEM, was statistically significant, *b* = –0.00003, *t*(198) = –2.00, *p* < 0.05.

**Table 2:** Coefficients and 95% confidence intervals of individual variables and two-way moderation for predicting gaming disorder (male sample).

Model summary	*R*	*R^2^*	*MSE*	*F*	*df*	*p*
	.3889	0.1513	8.7763	11.7627	3, 198	< 0.001
Model	*coeff*	*SE*	*t*	*p*	LLCI	ULCI
GT	0.0027	0.0009	2.8838	< 0.01	0.0009	0.0046
IEM	0.0982	0.0278	3.5352	< 0.001	0.0434	0.1529
GT × IEM	– 0.00003	0.00001	**–** 1.9996	< 0.05	– 0.00005	– 0.0000004

MMSE, Mean Squared Error; df, degrees of freedom; LLCI, lower level of confidence interval; ULCI, upper level of confidence interval; coeff, coefficient; SE, standard error; GT, gaming time; IEM, Immersion/escapism motive.

**Figure 1:**
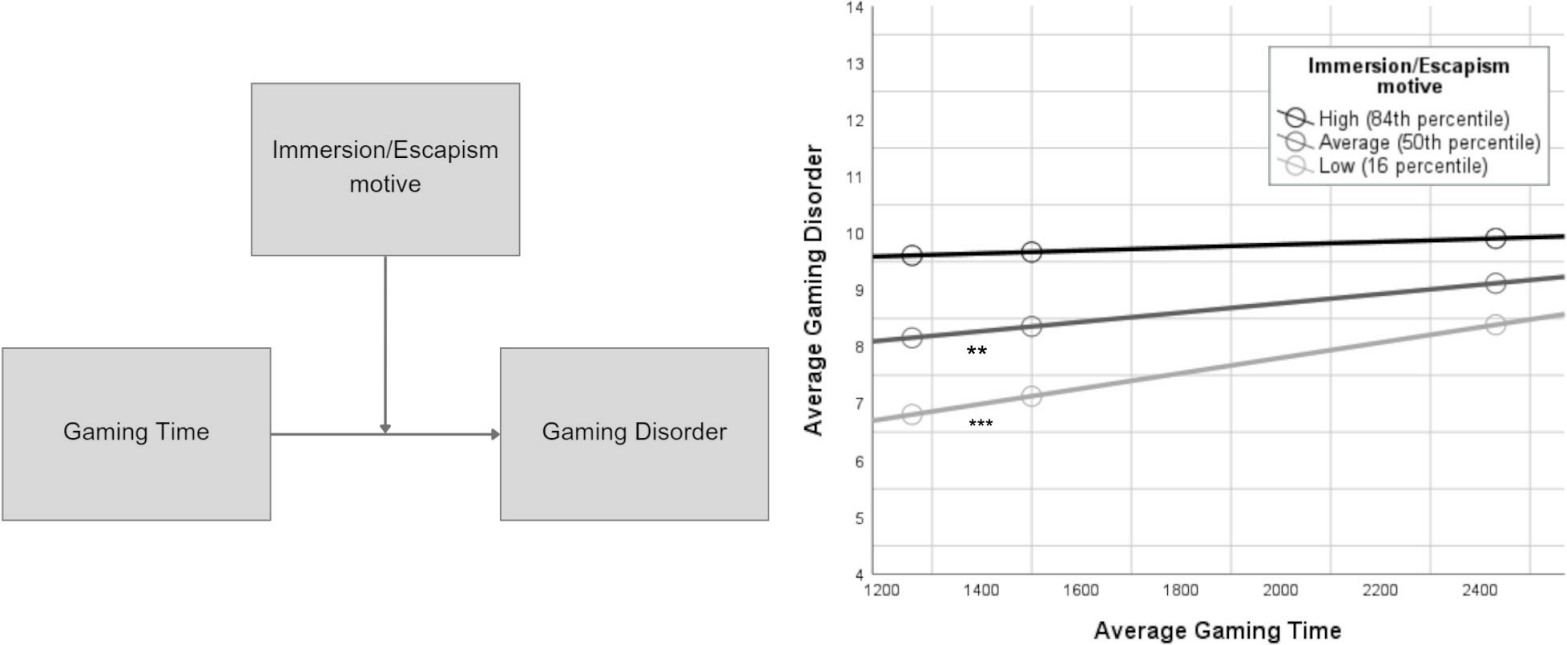
Moderation conceptual model and visual representation of gaming time's faciliatory effect on Gaming Disorder at high, average and low levels of immersion/escapism motive (two-way moderation). The faciliatory effect of immersion/escapism motive on the positive relationship between gaming time and Gaming Disorder was found significant at average (p = 0.01) and low (*p* < 0.001) levels of IEM, whereas it was not significant at high levels (p = 0.56). ***p < 0.001, **p < 0.01.

A calculation of the simple main effects (16th, 50th, and 84th percentiles) on the moderator revealed that only in the case of low (53.48) and moderate (74.00) levels of IEM, GT leads to a significant increase in GD risk, *b* = 0.001, *t*(198) = 3.78, *p* < 0.001 and *b* = 0.001, *t*(198) = 2.83, *p* < 0.01 respectively. For high (96.00) levels of IEM this effect was not significant, *b* = 0.0003, *t*(198) = 0.59, *p* = 0.56. These results are presented in **Table 3** and the visual representation is shown in **Figure 1**.

**Table 3:** Conditional effects of gaming time at values of immersion/escapism motive.

IEM	Effect	*SE*	*t*	*p*	LLCI	ULCI
53.48	0.0014	0.0004	3.7813	< 0.001	0.0006	0.0021
74.00	0.0008	0.0003	2.8257	< 0.01	0.0002	0.0014
96.00	0.0003	0.0004	0.5847	0.5594	**–** 0.0006	0.0011

IEM, Immersion/escapism motive; SE, standard error; LLCI, lower level of confidence interval; ULCI, upper level of confidence interval. The table presents IEM at 16th percentile (53.48), 50th percentile (74.00), and 84th percentile (96.00).

Additionally, the Johnson-Neyman technique was performed to determine specific values of the IEM at which the moderation occurred. It was found that when IEM levels were lower than 81.35, the moderation effect was significant. As IEM levels decreased, the relationship between GT and GD increased, with the lowest IEM level at 30, *b* = 0.002, *t*(198) = 3.32, *p* = 0.001.

Moderation analysis was also performed on a female sample (N = 92) but the effect was not statistically significant (**Supplementary Table S1** in the **Supplementary Materials**). However, further exploratory analyses were performed, and the linear regression analysis showed that IEM is a significant predictor of GD risk in this group. The results can be found in **Supplementary Table S2** in the **Supplementary Materials**.

## 4 Discussion

The results based on the responses of highly engaged male gamers shed new light on several important findings that contribute to the understanding of the factors associated with gaming disorder (GD). Specifically, we confirmed the role of escapism (IEM) as a moderator of the relationship between gaming time (GT) and the risk of GD in the sample of highly engaged male gamers. However, the detailed results are not entirely as expected.

Our results on the role of IEM in the GT-GD relationship correspond to previous ones, which advocate that escapism is a significant moderator of the relationship between GT and GD (36). However, the previous study has shown that 'GD symptoms were stronger among those with (…) higher (…) escapism scores' (36), while our study gave the opposite results. Specifically, the positive relationship between GT and GD was significant at low and moderate levels of IEM. In other words, longer gaming is more likely to co-exist with symptoms of GD only among low-to-moderate IEM individuals (in contrast to high-IEM). There might be a couple of explanations for these results. Individuals with low to moderate IEM might use gaming as a means to temporarily relieve them from life's difficulties, but not to the extent that it becomes their primary motivation or coping strategy. Nevertheless, it can still reinforce their gaming habits. The relief provided by gaming can turn problematic if it starts to disrupt other areas of their life. IEM closely aligns with Stenseng and colleagues' (40) concept of self-suppression escapism, which is linked to poor self-regulation (41). Consequently, individuals with low-to-moderate IEM might struggle with controlling their GT, potentially neglecting other activities or personal needs, which corresponds with the criteria for GD risk (1), thus, leading to IEM reinforcing the relationship between GT and GD.

In Koncz and colleagues' (36) study, escapism served as the moderator, whereas in the current study, IEM is the moderator. IEM encompasses a broader range of motives. This difference is crucial within the context of the Compensatory-Dissociative Online Gaming (C-DOG) model (42). According to the model, gaming behaviors lie on a continuum, with compensatory involvement characterized by a balanced relationship between the physical and virtual environments at one end, and dissociative involvement, marked by a separation between these environments to avoid emotional dysregulation and mental suffering, at the other.

Our study revealed that among participants with low to moderate IEM, more time spent gaming is indeed associated with an increased risk of GD. However, regardless of the combination of other parameters, it never exceeds the risk of GD presented by participants with high IEM. While our study did not directly explore dissociation, it can be speculated that participants with high IEM scores might engage in gaming dissociatively. High IEM scores encompass high levels of escapism, fantasy (playing games to immerse oneself in the game), introjected regulation (playing not to feel bad about oneself), coping (playing to de-stress), and identity (playing because games are an extension of oneself). When combined at high levels, these factors may align with the profile of dissociative involvement as described in the C-DOG model (42).

If this perspective is accurate, the C-DOG model could provide valuable context for understanding our findings. According to the model's authors, dissociative involvement is identified as the most maladaptive gaming pattern due to its association with a rigid separation between virtual and physical environments, and a pervasive denial of psychological needs. In such cases, the virtual self can become a defense mechanism against psychological disintegration, potentially resulting in compulsive gaming behaviors. Therefore, highly engaged gamers with elevated levels of IEM—whose profiles resemble dissociative involvement—may be at relatively equal risk of GD regardless of the total time spent gaming.

However, it's important to note that the above points are speculative. While IEM shows promise due to its multidimensional nature, it remains largely unexplored. The limited literature on this emerging concept highlights the need for further empirical research. Future studies could aim to explore how factors such as gaming duration and other behavioral patterns influence the risk of GD at various IEM levels, and why higher IEM might present a distinct risk profile compared to lower levels. This could be achieved by extending the range of variables considered to include those discussed in the limitations section of this study.

### 4.1 Gender differences

The moderation effect was not statistically significant for the female sample, which is consistent with the literature (36). Moreover, in the case of women highly involved in gaming, the results showed no significant correlation between GT and GD risk and a moderate positive correlation between IEM and GD risk. So, while the quantity of time spent gaming may not be a determining factor, the reasons for gaming seem to be more significant in the context of GD. It is further supported by the regression analysis which showed that IEM is a significant predictor of GD risk among this group. A possible explanation for these results is that after a certain level of involvement in gaming (at least 20 hours per week), GT ceases to play a role in the development of GD in the case of females, and the motivation to play becomes much more important. It corresponds to the results of the male sample, which showed that with high IEM motivation, the risk of GD is less dependent on GT.

However, in the context of the development of GD among highly committed gamers, women may show higher sensitivity to motivations while being less sensitive to GT than men. This may occur because women might be more focused on the social and emotional aspects of gaming over the sheer quantity of playtime. For women, gaming may serve as a means of social connection (43–45), emotional escape (46), or self-expression (45, 47, 48), factors which may outweigh the duration of gameplay in influencing the risk of GD. Additionally, societal expectations and gender norms may shape women's gaming behaviors differently (49), leading to distinct patterns of motivation and susceptibility to GD compared to men. Further research exploring these gender-specific dynamics is warranted to better understand the underlying mechanisms driving GD among highly committed female gamers.

### 4.2 Limitations

This study has several limitations that could be addressed in future research. Notably, we did not collect data on official mental health diagnoses, such as anxiety, depression, which could be significant for understanding the interplay among GT, IEM, and GD (20, 29, 50, 51). Including clinical assessments could provide a clearer view of how these conditions influence gaming behaviors. Additionally, we did not measure affect, self-regulation, or impulsiveness. These factors are important as they can significantly impact gaming habits and the risk of developing GD in the context of motives and gaming involvement (20, 26, 31, 52). Lastly, the study did not assess dissociation, a potential key factor in escapism and GD, according to the C-DOG model (42). Addressing these limitations in future studies will offer a more comprehensive understanding of the factors contributing to GD.

## 5 Conclusions

The findings underscore the multifaceted nature of GD, suggesting that it should not be solely attributed to the amount of time spent gaming. Instead, factors like gaming motivations play more significant roles in shaping gaming behaviors and their potential consequences, especially among highly engaged gamers. Future research should continue to investigate these complex interactions to develop comprehensive models of GD etiology, focusing on investigating why some individuals develop problematic gaming behaviors while others do not, despite similar GT. This approach allows us to shift focus from merely reducing GT to addressing the deeper motivations behind GD. By investigating the conditions under which GT becomes problematic, we can better identify when GT is associated with negative outcomes. This shift is critical because interventions that focus solely on decreasing GT might inadvertently stigmatize healthy gaming practices without addressing the core issues that drive GD. We advocate that effective therapeutic and preventive measures should not only target excessive gaming but also address the underlying motivations, such as IEM, that substantially contribute to the development of GD. Emphasizing a balanced approach to gaming that also considers motivational factors will lead to more effective prevention and intervention strategies, fostering a healthier gaming environment and addressing the root causes of GD.

## Data Availability

The datasets presented in this study can be found in online repositories. The names of the repository/repositories and accession number(s) can be found below: https://osf.io/7s9ar/.
