# Supplementary material for: High immersion/escapism motivation makes gaming disorder risk less dependent of playtime among highly engaged male gamers
Source: Front Psychiatry. 2024 Sep 25;15:1443091. doi: 10.3389/fpsyt.2024.1443091 (PMC11461240; doi:10.3389/fpsyt.2024.1443091)
Supplement: Supplementary file 1 [file Table1.docx]

# **Supplementary Materials**

## Participants selection details

The survey was conducted in January 2022, via the Internet, using the Qualtrics survey platform (https://qualtrics.com/). The study's URL was shared on gaming-related Polish groups on Facebook. There were two screening questions regarding the minimum age of 13 years and playing video games, and two attention checks in the survey.

A total of 1446 Polish-speaking participants took part in the study. Data from 68 were rejected because they did not meet the screening criteria. Additionally, 9 respondents who claimed to spend 24 or more hours per day gaming were not included in the analysis. This resulted in a sample of 1369 participants.

The gaming motivation tool used in the study (GMI) was designed based on highly engaged gamers who play at least 20 hours a week (Király et al., 2022). Therefore we decided to apply the same criteria in this study, selecting only highly engaged gamers who play at least 20 hours per week. The study by Koncz et al. (2022) showed that the escapism motive moderates the relationship between gaming time and gaming disorder only in the case of male players. We conducted the analyses on both gender samples, however the significant effect was only expected in the male sample.

## Statistical analysis (female sample)

The interactional influence between gaming time and IEM on GD was analyzed in the female sample, as shown in Table S1. The interaction term, GT × IEM, was not statistically significant, *b* = 0.00002, *t*(88) = 1.13, *p* = 0.26.

**Table S1**

*Coefficients and 95% confidence intervals of individual variables and two-way moderation for predicting Gaming Disorder (female sample)*

| Model summary | *R* | *R^2^* | *MSE* | *F* | *df* | *p* |
| --- | --- | --- | --- | --- | --- | --- |
|  | .3923 | 0.1539 | 0.025 | 5.3344 | 3, 88 | < 0.01 |
| Model | *coeff* | *SE* | *t* | *p* | LLCI | ULCI |
| GT | – 0.000188 | 0.000132 | – 1.426309 | 0.157317 | – 0.000451 | 0.000074 |
| IEM | – 0.000153 | 0.002824 | – 0.054262 | 0.95685 | – 0.005766 | 0.00546 |
| GT × IEM | 0.000002 | 0.000002 | 1.131815 | 0.260787 | – 0.000001 | 0.000005 |

*Notes*. MMSE: Mean Squared Error; df: degrees of freedom; LLCI: lower level of confidence interval; ULCI: upper level of confidence interval; coeff: coefficient; SE: standard error; GT: gaming time; IEM: Immersion/Escapism motive.

Simple linear regression analysis was used to verify whether Immersion/escapism motive significantly predicts gaming disorder risk in the female sample. The results suggested that Immersion/escapism motive explained 12% of the variance, *F*(1, 90) = 12.7, *p* < 0.001. Immersion/escapism motive significantly predicted gaming disorder risk in the sample, *ß* = 0.352, *t* = 3.564, *p* < 0.001. The results can be seen in the Table S2.

**Table S2**

*Results of linear regression analysis for the variable independent variable (Immersion/escapism motive) and dependent variable (gaming disorder risk)*

| *ß* | *b* | SE | R^2^ | F | *t* | *p* |
| --- | --- | --- | --- | --- | --- | --- |
| 0.352 | 0.003 | 0.001 | 0.124 | 12.700 | 3.564 | < 0.001 |

*Notes*. SE: standard error.

## Gaming Involvement - The scale of involvement in video games

Average weekly gaming time was assessed with two separate questions asking the exact minutes for an average weekday and weekend day. The two variables were combined during the analysis ([hours on an average weekday X 5] + [hours on an average weekend day X 2]). The instructions for the test subjects and the tool used are presented below.

How many MINUTES a day do you spend on the following activities?

|  | On a working day | On a weekend day |
| --- | --- | --- |
| 1. **Playing video games.** |  |  |
| 2. Thinking about video games. |  |  |
| 3. Reading guides, tutorials, reviews, theories, and additional lore related to video games. |  |  |
| 4. Watching video game streams and gameplays (including esports). |  |  |
| 5. Talking or texting about video games. |  |  |
| 6. Considering buying additional content and collectibles related to games (skins, gamepasses, ingame currency, figurines, t-shirts). |  |  |
